# Supplementary material for: Structure-based prediction of protein-nucleic acid binding using graph neural networks
Source: Biophys Rev. 2024 Jun 26;16(3):297–314. doi: 10.1007/s12551-024-01201-w (PMC11427629; doi:10.1007/s12551-024-01201-w)
Supplement: Supplementary file 1 — Supplementary file1 (PDF 1.25 MB) [file 12551_2024_1201_MOESM1_ESM.pdf]

Supplementary Data

Structure-based prediction of protein-nucleic acid binding using graph neural networks

Jared M. Sagendorf[1,6], Raktim Mitra[1], Jiawei Huang[1], Xiaojiang S. Chen[2,3], Remo Rohs[1,3,4,5,*]

[1]Department of Quantitative and Computational Biology, University of Southern California, Los Angeles, CA 90089, USA.

[2]Molecular and Computational Biology Section, Department of Biological Sciences, University of Southern California, Los Angeles, CA 90089, USA.

[3]Department of Chemistry, University of Southern California, Los Angeles, CA 90089, USA.

[4]Department of Physics and Astronomy, University of Southern California, Los Angeles, CA 90089, USA.

[5]Thomas Lord Department of Computer Science, University of Southern California, Los Angeles, CA 90089, USA.

[6]Present address: Department of Bioengineering and Therapeutic Sciences, University of California San Francisco, San Francisco, CA 94158, USA.

*Correspondence: rohs@usc.edu

Supplementary Tables 1–2; Supplementary Figures 1–5

Supplementary Tables

Feature Description(s)	Feature Abbreviations	Implementation	Type	Resolution
Electrostatic potential and surface normal derivative.	PH1, PH2	TABI-PB (Geng and Krasny 2013)	Electrostatic	Vertex
Atchley factors(Atchley et al. 2005) – summary of residue physicochemical properties by factor analysis.	AF1, AF2, AF3, AF4, AF5	PNAbind library	Chemical	Residue
Spatial aggregation propensity (Chennamsetty et al. 2010) – a measure of hydrophobicity.	SAP	PNAbind library	Chemical	Atom
Hydrogen bond donor/acceptor atoms.	HBA, HBD	PNAbind library	Chemical	Atom
Circular variance (Ceres et al. 2012) – a measure of accessibility defined over different distance scales.	CV1, CV2, CV3	PNAbind library	Geometric	Atom
Mean curvature – a measure of curvature which is positive for convex regions and negative for concave.	MC	libigl (Jacobson et al. 2018)	Geometric	Vertex
Heat kernel signature (Sun et al. 2009) – a shape descriptor based on eigenvectors of the Laplace-Beltrami operator.	HK1, HK2, HK3, HK4	Libigl	Geometric	Vertex
PSSM entry features produced by multiple sequence alignment against the UniRef90 database.	PSSM1–20	PSI-BLAST (Altschul et al. 1997)	MSA	Residue
Profile HMM features produce by HMM-HMM alignments against the UniRef30 database.	HMM1–30	HHblits (Remmert et al. 2012)	MSA	Residue

Supplementary Table 1: A complete list of vertex features used in our method. Feature abbreviations are shorthand for every feature name used in figures throughout the manuscript. Implementation is any external packages or software which PNAbind uses to implement the respective feature. The references under this column refer to the reference where the implementation was described. Type refers to which group a feature belongs to – electrostatic, chemical, geometric, or multiple-sequence alignment (MSA). Resolution refers to what structural resolution the feature is naturally defined for, which can be “Residue”, “Atom”, or “Vertex”.

DNA-136 dataset					
**AUROC**	**AUPRC**	**Precision**	**Recall**	**BA**	**MCC**
0.962	0.965	0.8234	0.884	0.848	0.6968

Supplementary Table 1: Results for distinguishing double-stranded DNA (dsDNA) binding proteins (dsDBP) from single-stranded DNA (ssDNA) binding proteins (ssDBP) based on datasets that are limited in size. DNA binding annotations at the level of specificity for dsDNA or ssDNA are sparse in the UniProt database, therefore a dataset of dsDBP (62 proteins) and ssDPB (74 proteins), DNA-136, was manually annotated based on available co-crystal complexes available in the PDB, and filtered/clustered using a very similar approach to how the larger datasets (DnP-6784, RnP-6046, RDP-6046) were constructed (described in main text). To make up for the small size of the resulting dataset, training data was augmented with additional residue-level binding site labels. This data augmentation produced highly accurate results and was validated using five-fold cross validation. The reported values are mean prediction metrics for the test folds. While we did not incorporate this data augmentation strategy in our other analysis, it suggests a promising strategy for future work in cases of smaller data sets.

Supplementary Figures

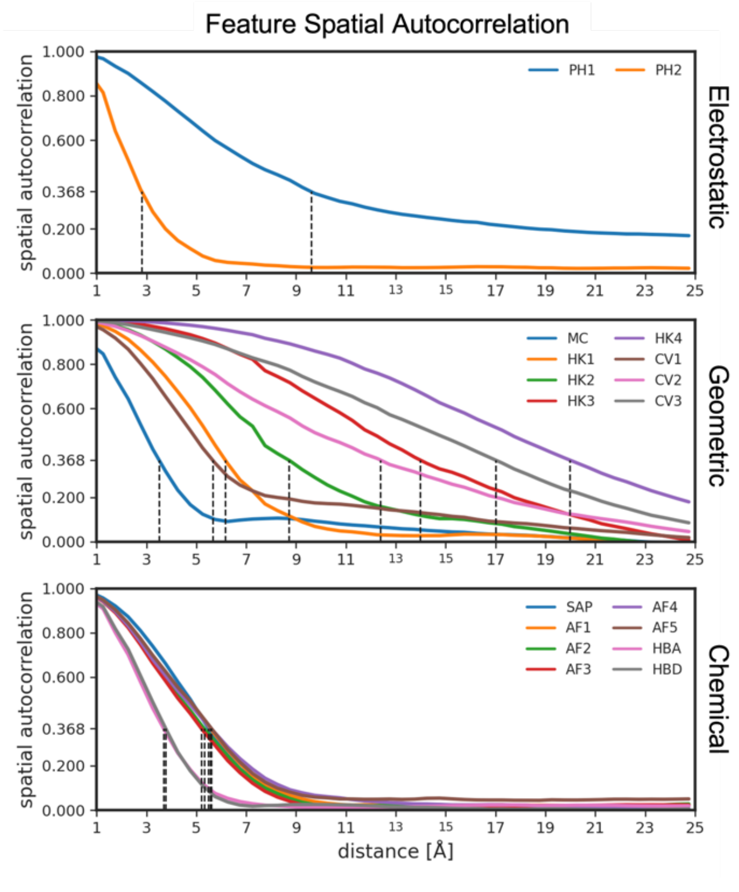

Supplementary Figure 1: Spatial autocorrelations for vertex features. Autocorrelations of eighteen structure-based features were computed using the Pearson correlation of feature channels between pairs of vertices within bins of increasing radial distance. Features which vary slowly over the mesh will have high autocorrelation over large distances, and features that vary rapidly have correlations that decay quickly. The dotted lines indicate the distance at which each feature autocorrelation drops to a value of $1/e$, corresponding to a characteristic distance scale for that feature.

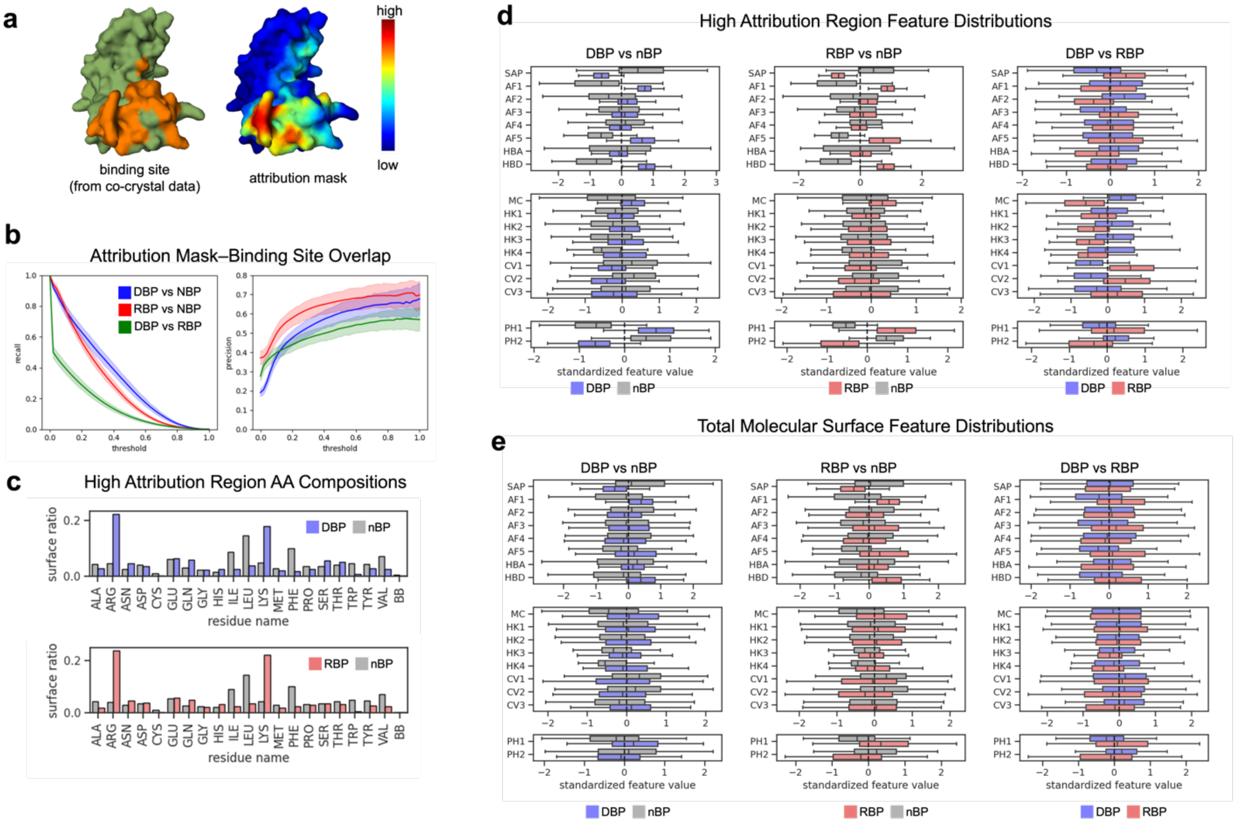

Supplementary Figure 2: Spatial attribution comparisons and correlation with binding sites. A Precision and recall curves computed from normalized spatial attribution masks and binding site labels transferred from proteins with experimental co-crystal structures available. **b** Proportion that each residue side chain and the peptide backbone (BB) contributes to the total surface area within high spatial attribution regions. The top plot was computed from the DBP versus nBP model and the bottom plot from the RBP versus nBP model. **c** Feature distributions within high spatial attribution regions for each classification model. **d** Feature distributions over the entire molecular surface for each classification model. Much less separation is seen in the distributions relative to those in panel **c**.

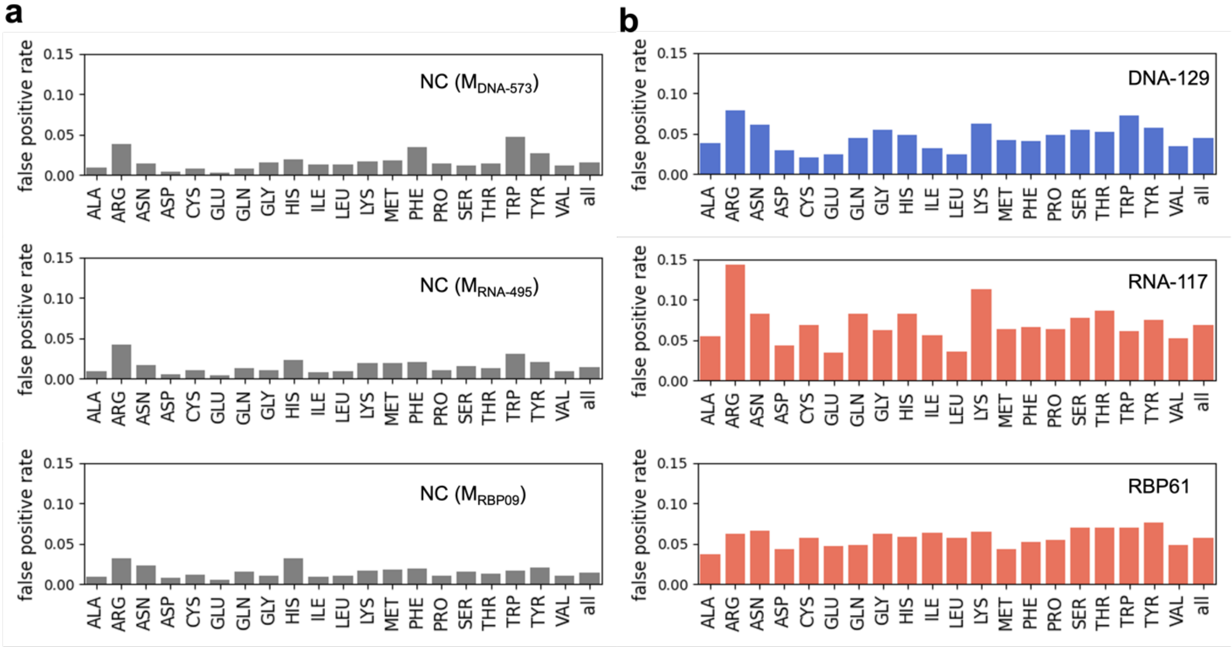

Supplementary Figure 3: Residue-level false positive rates for binding site prediction. a False positive rate for each residue type from the negative control (NC) dataset and the three binding site prediction models ($M_{DNA-573}$, $M_{RNA-495}$ M_{RBP09}). **B** False positive rates for each residue type from the benchmark test sets.

a

Experimental Training Datasets

Name	Type	# of target chains*	Positive Count	Negative Count
RBP09	RBP	808	33,160	208,931
DNA-573	DBP	573	14,479	145,404
RNA-495	RBP	495	14,609	122,290

Experimental Test Datasets

Name	Type	# of target chains*	Positive Count	Negative Count
RBP61	RBP	61	2974	8729
DNA-181	DBP	181	3208	72,050
DNA-129	DBP	129	2240	35,275
RNA-117	RBP	117	2031	35,314
NC	NBP	340	0	133,611

b

AlphaFold Datasets

Name	Total Structures	Positive Count	Negative Count
DnP-6784	6784	3392	3392
RnP-6046	6046	3392	2654
RDP-6046	6046	2654	3392

c

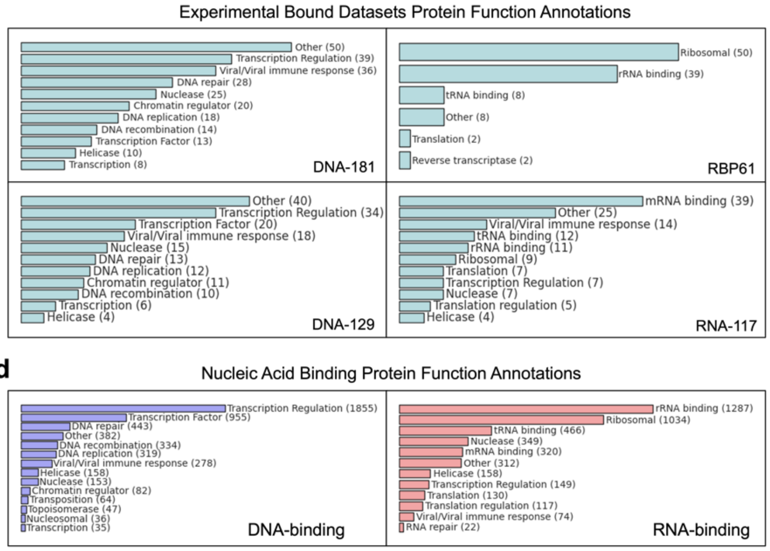

Supplementary Figure 4: Statistics and functional annotations of datasets used in this study. a Statistics for binding site prediction training and test sets containing experimentally determined protein structures. Positive count refers to the number of residues labeled as NA binding, and negative count is the number of solvent exposed residues that do not interaction with nucleic acids in the observed complex. **b** Statistics for binding function prediction datasets comprising protein structures predicted by AlphaFold2. Positive count refers to number of proteins labeled as the positive class (DNA binding for DnP-6784, RNA binding for RNB-6046 and RNA binding for RDP-6046) and negative count is the number of proteins labeled as the negative class. **c**–**d** Functional annotations derived from UniProt and Gene Ontology (Ashburner et al. 2000) molecular function annotations for proteins in the datasets shown in panels **a**–**b**.

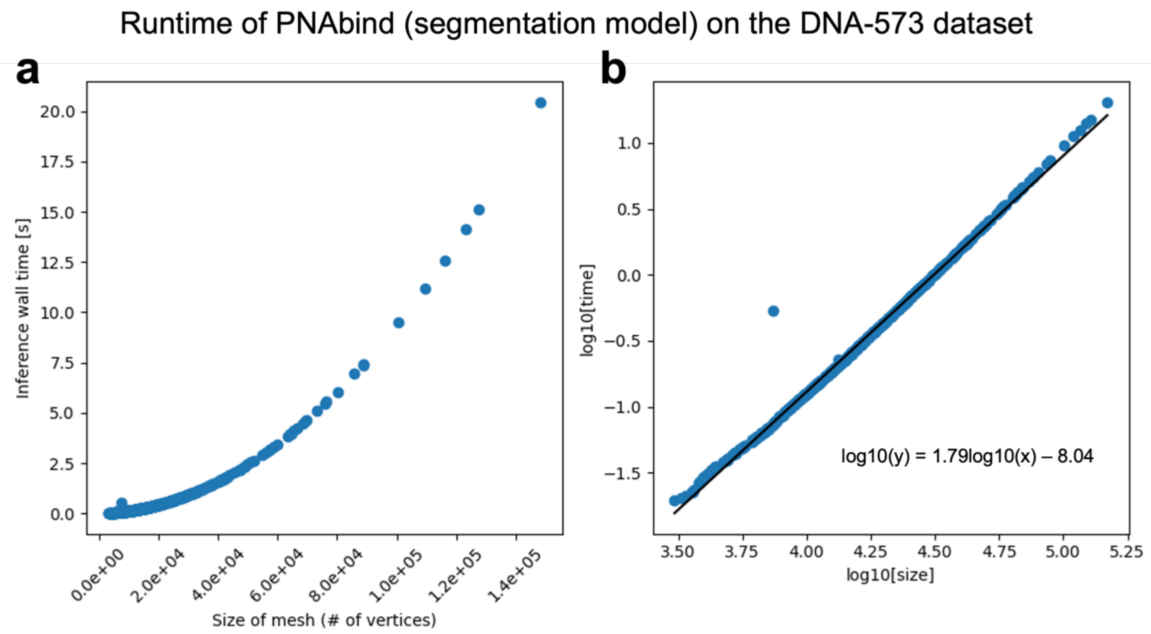

Supplementary Figure 5: Runtime of PNAbind. a Mesh size (number of vertices in the mesh) is plotted against the wall time for evaluation of a segmentation model (inference, not training). Each point is a single protein surface mesh, corresponding to a protein assembly in the DNA-573 training set. Inference can be performed on the vast majority of meshes in under 5s. Timing was performed on an NVIDIA Quadro RTX 5000 GPU. **b** The same plot as in a, but with logarithm scale. The time complexity is approximately quadratic in the number of vertices.

Supplementary References

Altschul, Stephen F., Thomas L. Madden, Alejandro A. Schäffer, Jinghui Zhang, Zheng Zhang, Webb Miller, and David J. Lipman. (1997) Gapped BLAST and PSI-BLAST: a new generation of protein database search programs. *Nucleic Acids Res.*, 25: 3389-402.

Ashburner, M., C. A. Ball, J. A. Blake, D. Botstein, H. Butler, J. M. Cherry, A. P. Davis, K. Dolinski, S. S. Dwight, J. T. Eppig, M. A. Harris, D. P. Hill, L. Issel-Tarver, A. Kasarskis, S. Lewis, J. C. Matese, J. E. Richardson, M. Ringwald, G. M. Rubin, and G. Sherlock. (2000) Gene ontology: tool for the unification of biology. The Gene Ontology Consortium. *Nat. Genetics*, 25: 25-9.

Atchley, William R., Jieping Zhao, Andrew D. Fernandes, and Tanja Drüke. (2005) Solving the protein sequence metric problem. *Proc. Natl. Acad. Sci. USA*, 102: 6395-400.

Ceres, Nicoletta, Marco Pasi, and Richard Lavery. (2012) A Protein Solvation Model Based on Residue Burial. *J. Chem. Theor. Comput.*, 8: 2141-44.

Chennamsetty, N., V. Voynov, V. Kayser, B. Helk, and B. L. Trout. (2010) Prediction of aggregation prone regions of therapeutic proteins. *J. Phys. Chem. B*, 114: 6614-24.

Geng, Weihua, and Robert Krasny. (2013) A treecode-accelerated boundary integral Poisson-Boltzmann solver for electrostatics of solvated biomolecules. *J. Comput. Phys.* 247: 62-78.

Jacobson, Alec, Daniele Panozzo, et al. (2018) libigl: A simple C++ geometry processing library. In https://libigl.github.io.

Lam, Jordy Homing, Yu Li, Lizhe Zhu, Ramzan Umarov, Hanlun Jiang, Amélie Héliou, Fu Kit Sheong, Tianyun Liu, Yongkang Long, Yunfei Li, Liang Fang, Russ B. Altman, Wei Chen, Xuhui Huang, and Xin Gao. (2019) A deep learning framework to predict binding preference of RNA constituents on protein surface. *Nat. Commun.*, 10: 4941.

Li, Pengpai, and Zhi-Ping Liu. (2022) PST-PRNA: prediction of RNA-binding sites using protein surface topography and deep learning. *Bioinformatics*, 38: 2162-68.

Li, Pengpai, and Zhi-Ping Liu. (2023) GeoBind: segmentation of nucleic acid binding interface on protein surface with geometric deep learning. *Nucleic Acids Res.*, 51: e60.
Remmert, Michael, Andreas Biegert, Andreas Hauser, and Johannes Söding. (2012) HHblits: lightning-fast iterative protein sequence searching by HMM-HMM alignment. *Nat. Methods*, 9: 173-75.
Roche, Rahmatullah, Bernard Moussad, Md Hossain Shuvo, Sumit Tarafder, and Debswapna Bhattacharya. (2024) EquiPNAS: improved protein–nucleic acid binding site prediction using protein-language-model-informed equivariant deep graph neural networks. *Nucleic Acids Res.*, 52: e27.
Sun, Jian, Maks Ovsjanikov, and Leonidas Guibas. (2009) A Concise and Provably Informative Multi-Scale Signature Based on Heat Diffusion. *Comp. Graph. Forum*, 28: 1383-92.
Xia, Ying, Chun-Qiu Xia, Xiaoyong Pan, and Hong-Bin Shen. (2021) GraphBind: protein-structural context embedded rules learned by hierarchical graph neural networks for recognizing nucleic-acid-binding residues. *Nucleic Acids Res.*, 49: e51.
Yuan, Qianmu, Sheng Chen, Jiahua Rao, Shuangjia Zheng, Huiying Zhao, and Yuedong Yang. (2022) AlphaFold2-aware protein-DNA binding site prediction using graph transformer. *Brief. Bioinform.*, 23: bbab564.
